# Influence of Oligopeptide Length and Distribution on Polyisoprene Properties

**DOI:** 10.3390/polym13244408

**Published:** 2021-12-15

**Authors:** Chang-Cheng Wang, Rong Zhang, Shiqi Li, Guangsu Huang, Maozhu Tang, Yun-Xiang Xu

**Affiliations:** College of Polymer Science & Engineering, State Key Laboratory of Polymer Materials Engineering, Sichuan University, Chengdu 610065, China; 2019223090113@stu.scu.edu.cn (C.-C.W.); 18980908620@163.com (R.Z.); 18702882636@163.com (S.L.); huangguangsu@scu.edu.cn (G.H.)

**Keywords:** polyisoprene, oligopeptide length, distribution, terminal block

## Abstract

The tuning of binding modes of polar groups is the key step to mimicking the structure and properties of natural rubber through the molecular design of synthetic polyisoprenes. Herein, the ordering and binding distances of oligopeptides could be altered systematically by changing their lengths and distribution along the polyisoprene chain, which impose huge impacts on the mechanical properties and chain dynamics of green rubber. In detail, a series of peptide-functionalized polyisoprenes with terminal blocks (B-2A-PIP, B-3A-PIP) or random sequences (R-2A-PIP, R-3A-PIP) are fabricated by using dipeptides (2A) or tripeptides (3A) as crosslinkers to explore the mechanism of terminal interaction on mechanism properties and chain dynamics. B-4A-PIP and R-4A-PIP served as control samples. It is found that the increased oligopeptide length and the block distribution improves the mechanical properties and confine the chain movement by elevate the contents of ordered and compact microstructures, which is indicated by XRD, broadband dielectric spectroscopy (BDS) and consistent with the result of molecular dynamics simulation. New relaxation signals belonging to oligopeptide aggregates are found which showed elevated dielectric strengths upon temperatures increase. Additionally, it also reveals that the binding modes of oligopeptide do not significantly influence the entanglements of polyisoprene.

## 1. Introduction

As an important strategic material, natural rubber (NR) is used in a wide range of livelihood and military applications, such as tires, seals, and aerospace applications. This is due to its excellent comprehensive performance, such as high strength, high toughness, high abrasion resistance, tear resistance, and wet skid resistance [1,2,3]. However, the natural conditions of the origin greatly limit the quality and yield of natural rubber. Therefore, it is necessary to find alternatives to natural rubber. IR is the most promising synthetic rubber to replace natural rubber, which has similar chemical composition and cis content with NR [4]. Nevertheless, the comprehensive properties of IR are quite inferior to those of NR, such as tensile strength and fatigue resistance [5,6,7]. Practically, the properties of NR are not only related to the cis content and molecular weight, but also closely related to their terminal structures [8]. In particular, the α-terminal and ω-terminal consist of phospholipids or non-covalently linked proteins, respectively [9]. Different polar terminal structures make distinct contribution on the mechanical properties. However, so far it is relatively challenging to reverse engineer the terminal structures of NR by synthetic approach due to their complicated components. For example, several types of proteins are found in NR and each type is one kind of huge molecule. Therefore, to mimic the terminal structures of NR from the principal level, simplified system is urgently required.

It is generally assumed that the key functions of terminal structures of NR consists of two factors [10]. One is the formation of branching structures which elevated the entanglement of polyisoprene chains, and another one is the dissociation of noncovalent interactions to dissipate energy which are both closely related to the terminal supramolecular interactions. However, although a great deal of research has been conducted focused on the mechanism of supramolecular interactions on comprehensive properties, the support regarding terminal interactions such as NR is rare [11,12]. Moreover, the contributions of terminal groups are generally verified in tensile strength [13], dimensional stability [14], shape memory [15] and so on because of their unique structures. Thus, to construct high performance elastomers, it is valuable to establish the structure–property relationship of terminal interactions on high performance elastomers via precisely tuneable systems.

As a commonly utilized self-assembly tool, oligopeptide has the potential to provide aggregates with tuneable supramolecular interactions and self-sorting properties. If proper terminal structures and oligopeptide structures are selected, precise terminal supramolecular interactions between different polymer chains would occur and their assembly or dissociation could be manipulated. Specifically, the hydrogen bonding numbers of oligopeptide units can be expediently tuned by changing the oligopeptide backbone length [16], which significantly tunes the supramolecular binding strength and impact the elastomer properties. For instance, Jia et al. [17] synthesized a series of star-block copolymers comprising of polyisobutylene stars and oligo(β-alanine) end segments with different length. β-alanine dimer is partially hydrogen-bonded, while the trimer, tetramer and pentamer are fully hydrogen-bonded and form β-sheets. As the oligopeptide length increased, the melting temperatures of crystalline domains raised and eventually increased the rubber elastic moduli.

In addition, the oligopeptides can form aggregates and affect the dynamics of the multiscale units of the polymer. To better understand the structure–property relationships on the molecular unit concepts, the molecular dynamics are necessary. Dielectric spectroscopy is a powerful tool for detecting multi-level molecular units relaxation, which can easily allow to cover 10^−6^–10^12^ Hz in frequency [18]. In the present work, we use cis-1,4-PI as the bulk material. Owing to the asymmetrical structure, cis-1,4-PI has a dipole moment both parallel and perpendicular to the chain contour [19]. Usually, it presents two relaxation modes under an external electric field, defined as segmental mode (SM) relaxation and normal mode (NM) relaxation, respectively. SM relaxation is caused by the perpendicular component of the dipole, and NM relaxation is caused by the parallel component [20]. Valid information can be obtained from the changes of multi-level molecular units relaxation, and therefore, dielectric spectroscopy will contributes to a better understanding of the structure–property relationships.

**Scheme 1:**
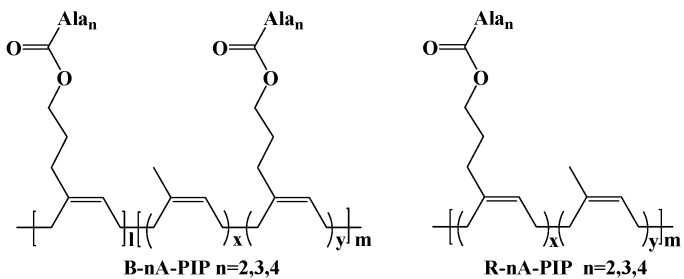
Chemical structures of oligopeptide functionalized polyisoprene with terminal blocks (B-nA-PIP, n = 2, 3, 4) or random sequences (R-nA-PIP, n = 2, 3, 4).

In our previous research of mimicking the terminal structure of natural rubber, techniques to prepare terminally oligopeptide functionalized polyisoprene have been developed [21]. However, how to tune the supramolecular binding strength of terminal structures and their principle to maintain terminal interactions need to be further explored. In this paper, besides the previously prepared B-4A-PIP and R-4A-PIP, dipeptide and tripeptide units were grafted onto hydroxyl-functionalized polyisoprenes B-OH-PIP with terminal block and R-OH-PIP with random sequence, producing B-2A-PIP/B-3A-PIP and R-2A-PIP/R-3A-PIP, respectively (Scheme 1). It is found oligopeptide backbone lengths influence the mechanical properties, relaxation processes of elastomers and the self-assembly mode of oligopeptide themselves because of different terminal supramolecular strength and β-sheet aggregation. Furthermore, the terminal block of oligopeptide will self-sort to form stronger terminal interactions regardless of the peptide length, which provide a robust tool to construct the mimicking terminal structures of NR. 

## 2. Materials and Methods

### 2.1. Materials

B-OH-PIP (Mn = 9.2 × 10^5^) and R-OH-PIP (Mn = 8.2 × 10^5^) were synthesized by the stereoselective coordination polymerization in the glove box according to our previous method [21]. The average number of hydroxyl groups in the terminal block of B-OH-PIP copolymer is approximately forty, while about one hundred hydroxyls are randomly dispersed in the rest polyisoprene chain. For R-OH-PIP, there are about 130 hydroxyl groups randomly dispersed in the polyisoprene chain. Additionally, B-4A-PIP and R-4A-PIP are prepared according to our previous study [21]. Tetrahydrofuran was refluxed over sodium/diphenylketone under nitrogen and then distilled before use. Other chemicals, unless otherwise specified, were purchased from Aldrich (Aldrich, Shanghai, China) and used as received.

### 2.2. Synthesis of Samples

#### 2.2.1. B-2A-PIP

Step 1: The preparation of 2Ala-OCH2Ph. Boc-2Ala-OCH2Ph was synthesized according to the reported procedure [22]. Boc-2Ala-OCH2Ph (399 mg, 1.14 mmol) was resolved in the dichloromethane (3 mL) for 10 min and then trifluoroacetic acid (TFA, 7 mL) was slowly added into the suspension at the room temperature. The solution was stirred overnight and concentrated by rotary evaporation. The crude product 2Ala-OCH2Ph was then used without further purification. Step 2: The preparation of B-DSC-PIP. N,N′-disuccinimidyl carbonate (76.8 g, 300 mmol) and 4-dimethylaminopyridine (37.0 g, 300 mmol) were resolved in the mixed solution of dry THF (3000 mL) and DMF (1400 mL) in the three-neck bottle while B-OH-PIP (3.0 g) was resolved in dry THF (300 mL) in the flask. Secondly, the latter was slowly added into the mixed solution at 0 °C. The reaction was stirred at the room temperature for 8 h. The mixture was evaporated and poured into acetone to get the precipitate B-DSC-PIP. The crude grey product was collected (2.98 g, 99.3%) and the conversion ratio from hydroxyl group to carbonate group is detected with ^1^H-NMR, which is 99.4%, as shown in Figure S1. ^1^H NMR (400 MHz, Chloroform-d) δ: 5.12 (t, 2H), 4.29 (t, 2H), 2.83 (s, 4H), 2.04 (m, 10H), 1.83 (m, 2H), 1.68 (s, 3H). Step3. The synthesis of B-2A-PIP. 2Ala-OCH2Ph (1.14 mmol) and diisopropylethylamine (DIEA, 5.5 mL) were resolved in the mixed solution of THF (150 mL) and water (14 mL) in the three-neck bottle while B-DSC-PIP (1.5 g) was resolved in dry THF (300 mL) in the flask. Secondly, the latter was slowly added into the mixed solution at 0 °C. The reaction was stirred at the room temperature for 8 h. The mixture was evaporated and poured into water to get the precipitate B-2A-PIP. The precipitate was washed with water for three times and the final product was dried in vacuum oven at 45 °C to afford a gray elastomer (1.46 g, 97.3%). The conversion ratio from carbonate group to dipeptide is detected with ^1^H-NMR, which is 99.6%, as shown in Figure S2. ^1^H NMR (400 MHz, Chloroform-d) δ: 7.34 (s, 5H), 5.12 (t, 2H), 4.61 (m, 1H), 4.21 (m, 1H), 4.04 (t, 2H), 2.04 (m, 10H), 1.83 (m, 2H), 1.68 (s, 3H).

#### 2.2.2. Synthesis of B-3A-PIP

Step 1: The preparation of 3Ala-OCH2Ph. Boc-3Ala-OCH2Ph was synthesized according to the reported procedures [22]. Boc-3Ala-OCH2Ph (480 mg, 1.14 mmol) was resolved in the dichloromethane (3 mL) for 10 min and then trifluoroacetic acid (TFA, 7 mL) was slowly added into the suspension at the room temperature. The solution was stirred overnight and concentrated by rotary evaporation. The crude product was then used without further purification. Step 2: The synthesis of B-3A-PIP. 3Ala-OCH2Ph (1.14 mmol) and diisopropylethylamine (DIEA, 5.5 mL) were resolved in the mixed solution of THF (150 mL) and water (14 mL) in the three-neck bottle while B-DSC-PIP (1.48 g) was resolved in dry THF (300 mL) in the flask. Secondly, the latter was slowly added into the mixed solution at 0 °C. The reaction was stirred at the room temperature for 8 h. The mixture was evaporated and poured into water to get the precipitate B-3A-PIP. The precipitate was washed with water for three times and the final product was dried in vacuum oven at 45 °C to afford a gray elastomer (1.46 g, 98.6%). The conversion ratio from carbonate group to tripeptide is detected with ^1^H-NMR, which is 99.6%, as shown in Figure S3. ^1^H NMR (400 MHz, Chloroform-d) δ: 7.34 (s, 5H), 5.12 (t, 2H), 4.61 (m, 1H), 4.21 (m, 1H), 4.04 (t, 2H), 2.04 (m, 10H), 1.83 (m, 2H), 1.68 (s, 3H).

#### 2.2.3. Synthesis of R-2A-PIP

Step 1: The preparation of R-DSC-PIP. N, N′-disuccinimidyl carbonate (76.8 g, 300 mmol) and 4-dimethylaminopyridine (37.0 g, 300 mmol) were resolved in the mixed solution of dry THF (3000 mL) and DMF (1400 mL) in the three-neck bottle while R-OH-PIP (3.0 g) was resolved in dry THF (300 mL) in the flask. Secondly, the latter was slowly added into the mixed solution at 0 °C. The reaction was stirred at the room temperature for 8 h. The mixture was evaporated and poured into acetone to get the precipitate R-DSC-PIP. The crude grey product was collected (2.96 g, 98.7%) and the conversion ratio from hydroxyl group to carbonate group is detected with ^1^H-NMR, which is 97.8%, as shown in Figure S4. ^1^H NMR (400 MHz, Chloroform-d) δ: 5.12 (t, 2H), 4.29 (t, 2H), 2.83 (s, 4H), 2.04 (m, 10H), 1.83 (m, 2H), 1.67 (s, 3H). Step 2: The synthesis of R-2A-PIP. 2Ala-OCH2Ph (1.14 mmol) and diisopropylethylamine (DIEA, 5.5 mL) were resolved in the mixed solution of THF (150 mL) and water (14 mL) in the three-neck bottle while R-DSC-PIP (1.5 g) was resolved in dry THF (300 mL) in the flask. Secondly, the latter was slowly added into the mixed solution at 0 °C. The reaction was stirred at the room temperature for 8 h. The mixture was evaporated and poured into water to get the precipitate R-2A-PIP. The precipitate was washed with water for three times and the final product was dried in vacuum oven at 45 °C to afford a gray elastomer (1.48 g, 98.7%). The conversion ratio from carbonate group to dipeptide is detected with ^1^H-NMR, which is 98.3%, as shown in Figure S5. ^1^H NMR (400 MHz, Chloroform-d) δ: 7.34 (s, 5H), 5.12 (t, 2H), 4.61 (m, 1H), 4.21 (m, 1H), 4.04 (t, 2H), 2.04 (m, 10H), 1.83 (m, 2H), 1.67 (s, 3H).

#### 2.2.4. Synthesis of R-3A-PIP

3Ala-OCH2Ph (1.14 mmol) and diisopropylethylamine (DIEA, 5.5 mL) were resolved in the mixed solution of THF (150 mL) and water (14 mL) in the three-neck bottle while R-DSC-PIP (1.5 g) was resolved in dry THF (300 mL) in the flask. Secondly, the latter was slowly added into the mixed solution at 0 °C. The reaction was stirred at the room temperature for 8 h. The mixture was evaporated and poured into water to get the precipitate R-3A-PIP. The precipitate was washed with water for three times and the final product was dried in vacuum oven at 45 °C to afford a gray elastomer (1.43 g, 97.9%). The conversion ratio from carbonate group to tripeptide is detected with ^1^H-NMR, which is 99.6%, as shown in Figure S6. ^1^H NMR (400 MHz, Chloroform-d) δ: 7.34 (s, 5H), 5.12 (t, 2H), 4.58 (m, 1H), 4.45 (m, 1H), 4.20 (m, 1H), 4.05 (t, 2H), 2.04 (m, 10H), 1.83 (m, 2H), 1.67 (s, 3H).

### 2.3. Sample Molding Process

Each product (1.4 g) was resolved in toluene (70 mL) and the solution slowly volatilized in the fume hood to obtain a flat membrane. The product was then moved into the vacuum oven at 40 °C for 12 h to remove the solvent. The species were cut from the membrane according to requirements of different measurements. As a representative, a picture of the sample used for uniaxial tensile testing is shown in Figure S7.

### 2.4. Characterization

The Bruker ASCEND 400 spectrometer (Bruker, Karlsruhe, Germany) was used to collecting the ^1^H NMR spectra, operating at 400 MHz. The samples are resolved in deuterated chloroform solution with TMS as reference. The universal testing machine Instron 5966 (Instron, Boston, MA, USA) was used to measure mechanical properties at 25 °C. The tensile speed is 100 mm/min. The specimen was cut into a dumbbell shaped thin strip with dimension of 35 mm × 2 mm × 1 mm. For each data, the strength was calculated from three measurements and taken from the average value. The Nicolet iS10 (Nicolet, Waltham, MA, USA) was used to measure FTIR spectra in the range of 4000–400 cm^−1^. The Rigaku X-ray diffractometer (Ultima IV, Akishima-shi, Japan) were used to carry X-ray diffraction experiments with parallel beam optics attachment.

Novocontrol Concept 50 system (Novocontrol GmbH, Montabaur, Germany) was carried on dielectric measurements with Alpha impedance analyzer and Quatro Cryosystem temperature control over the frequency range of 10^−1^–10^7^ Hz. The measured film was about 1 mm thickness and placed between two parallel electrodes with 10 mm diameter. The measured temperatures were from −70 °C to 80 °C at the step of 10 °C. The result were fitted using the Havriliak–Negami function with a conductivity term [23]. In this model, the frequency dependence of the dieletric complex (ε*) can be described by the equation:

(1) $$\varepsilon^{*}\left(\omega \right)=\varepsilon_{\infty }+\frac{\Delta \varepsilon }{{\left[1+{\left(i\omega \tau_{HN}\right)}^{\alpha }\right]}^{\beta }}$$

where Δ*ε* = *ε*_s_ − *ε*_∞_ is the dielectric strength, *ε*_s_ and *ε*_∞_ are the relaxed and unrelaxed values of dielectric constant, the parameters *α* and *β* (0 < *α*, *αβ* ≤ 1) define the symmetrical and asymmetrical broadening of the loss peak, and *τ_HN_* is the characteristic relaxation time. The relation between *τ_HN_* and *τ*_max_ is given by Refs [24,25].

(2) $$\tau_{\max}=\tau_{HN}{\left[\sin\frac{\pi \alpha \beta }{2\left(1+\beta \right)}\right]}^{\frac{1}{\alpha }}{\left[\sin\frac{\pi \alpha }{2\left(1+\beta \right)}\right]}^{-\frac{1}{\alpha }}$$

This characteristic relaxation time (*τ*_max_) obtained from the *HN* equation fit can be correlated with the temperature through the Vogel–Fulcher–Tamman (VFT) equation [26,27]:

(3) $$\;\;\;\;\;\;\;\tau_{\max}=\tau_{0}\exp\left(\frac{B}{T-T_{0}}\right)$$

where *τ*_0_ and *B* are empirical parameters and *T*_0_ is the so-called Vogel temperature.

The polymer models were constructed using the polymer builder of Materials Studio package. The GROMACS [28,29,30,31,32] version 2018.3 was used to carry further full-atomistic classical molecular dynamics simulations. The forcefield used for this study was the OPLS all-atom (OPLSAA) force field [33,34,35,36], considering its wide applicability in different polymer systems. The timestep used for all simulations was taken as 1 fs. VMD [37] was used to visualize and analyze various properties based on MD trajectories saved every 5000 steps (5 ps). The equilibrated systems have densities of 0.890 ± 0.003 g/cm^3^, 0.899 ± 0.003 g/cm^3^ and 0.873 ± 0.017 g/cm^3^, for B-4A-PIP, B-3A-PIP and B-2A-PIP, respectively.

## 3. Results and Discussion

### 3.1. Mechanical Properties and Secondary Structure Analysis

**Figure 1:**
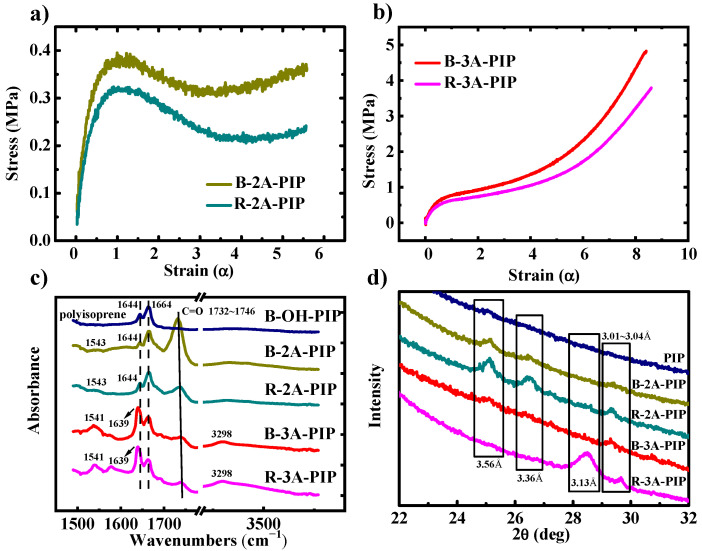
Stress–strain curves as a function of strain of (**a**) B-2A-PIP and R-2A-PIP; (**b**) B-3A-PIP and R-3A-PIP at the tensile rate of 100 mm/min. (**c**) FTIR spectra and (**d**) X-ray diffraction spectra of B-2A-PIP, R-2A-PIP, B-3A-PIP and R-3A-PIP.

B-OH-PIP and R-OH-PIP were decorated with dipeptides and tripeptides, producing B-2A-PIP/ B-3A-PIP, R-2A-PIP/ R-3A-PIP, respectively. The synthetic routes are shown in Figure S8. B-4A-PIP and R-4A-PIP serve as control samples. All copolymers have similar molecular weights and amounts of polar groups. The tensile strength and strain at break of B-2A-PIP and R-2A-PIP are relatively weak compared to those of B-3A-PIP and R-3A-PIP (Figure 1a), but slightly higher than those of B-OH-PIP and R-OH-PIP (Figure S9), respectively. The limited effect of dipeptide is ascribed to its weak binding strength. Compared to dipeptide counterparts, the mechanical properties of B-3A-PIP and R-3A-PIP are remarkably heightened, as shown in Figure 1b. Moreover, the mechanical properties of B-4A-PIP and R-4A-PIP are further increased (Figure S10a), revealing the significant influence of oligopeptide length on the mechanical properties of polyisoprene. Generally, in the aspect of the effect of peptide distribution, the sample with terminal block showed enhanced yield stress and tensile stress at break and increased length of oligopeptide improved the mechanical properties of elastomers. This indicated increased terminal supramolecular interactions produce certain positive role to mechanical properties of elastomers.

Then, FT-IR experiments were further carried out to reveal their difference on the secondary structures of polyisoprene (Figure 1c). Two characteristic bonds at 1644 cm^−1^ and 1664 cm^−1^ are observed for all the samples, attributing to the C=C stretching vibration of polyisoprene [38]. Importantly, obvious β-sheets structures were found at 1531 cm^−1^, 1632 cm^−1^ and 1706 cm^−1^ in B-4A-PIP and R-4A-PIP (Figure S10b). However, B-2A-PIP and R-2A-PIP give weak amide II absorption at 1543 cm^−1^ and overlapping amide I absorption at 1644 cm^−1^, corresponding to the random coils [39]. Random coils and extended chain structures of B-3A-PIP and R-3A-PIP are also observed as indicated by the existence of bands at 1541 cm^−1^ (amide II) and at 1639 cm^−1^ (amide I) [39]. The terminal sheet structures are closely related to their corresponding mechanical strength.

Thus, X-ray diffraction experiments were performed to further analyze the structural information of β-sheet (Figure 1d). The diffraction peaks with d spacing of 3.01~3.04 Å are assigned to the hydrogen spacing of β-sheet structures [40,41]. Typically, the augment of hydrogen bonds spacing [42,43] usually indicated weakened binding of hydrogen bonds. Hence, a second reflection around a spacing of 3.13 Å should be attributed to the hydrogen spacing in extended chain structures whose binding strength of hydrogen bonding aggregations is weaker than that of β-sheet structures. Additionally, the reflections at a spacing of 3.56 Å and 3.36 Å should be ascribed to the hydrogen spacing in loosely built β-sheets. In our research, the diffraction peaks of B-4A-PIP assigned to β-sheet and extended chain structures are obvious and sharper than R-4A-PIP (Figure S10c), while the diffraction peak of R-3A-PIP assigned to extended chain structures is also sharp. Additionally, the others are relatively weak. Especially, the diffraction peaks of B-2A-PIP and R-2A-PIP assigned to β-sheets and extended chain structures are both quite weak, while the diffraction peaks assigned to loosely built β-sheets are sharp. It has been reported that the increased oligopeptide length facilitates the formation of ordered and compact structures. However, as the aspect of distribution, the influence of terminal oligopeptide on the secondary structures are not consistent. The oligopeptide aggregates in R-2A-PIP and R-3A-PIP formed more ordered structures than those in B-2A-PIP and B-3A-PIP, mainly reflected in the more pronounced diffraction peaks of loosely built β-sheets and extended chain structures in R-2A-PIP and R-3A-PIP. That is, although the random samples are more ordered, loose aggregates dominate in these ordered structures, while compact aggregates dominate in the block samples (include B-4A-PIP and R-4A-PIP, as shown in Figure S10). Combined with the fact that the block samples have better mechanical properties than the random samples, it seems to indicate that the compact aggregates have a greater effect on the mechanical properties than the loose aggregates.

### 3.2. Molecular Dynamics-SM Relaxation, NM Relaxation and α′ Relaxation

**Figure 2:**
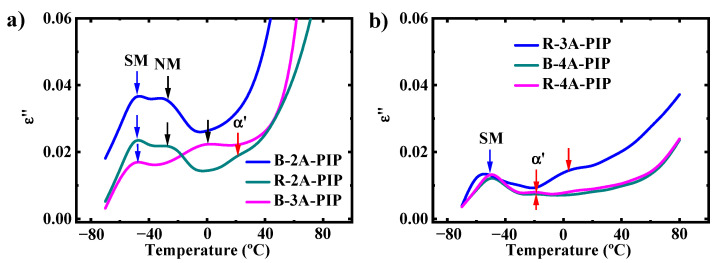
Dielectric loss *ε*″ for different copolymers as a function of the temperature at 6.9 Hz ((**a**) B-2A-PIP, R-2A-PIP, B-3A-PIP and (**b**) R-3A-PIP, B-4A-PIP, R-4A-PIP).

To explore the influence of terminal oligopeptide and its length on the chain dynamics of copolymers, broadband dielectric spectroscopy (BDS) was conducted. The temperature dependences of dielectric spectroscopy at 6.9 Hz are shown in Figure 2. A low-temperature relaxation just above the glass transition temperature (Tg~−65 °C), which is originated from local motions of the perpendicular dipole moment and assigned as the segmental-mode process (SM) [44,45] (indicated by blue arrows in Figure 2). However, there are no significant differences in the SM relaxation temperatures among these systems. A second broader relaxation peak indicated by black arrows should be assigned to the normal-mode process (NM), which corresponds to the motions of the entire chains [46,47]. The NM relaxation peaks of B-2A-PIP and R-2A-PIP are detected at −30 °C. For the B-3A-PIP sample, its NM relaxation peak shifts toward higher temperature and is detected at 0 °C. Notably, the NM relaxation peaks of R-3A-PIP, B-4A-PIP and R-4A-PIP disappear, maybe due to their stable network in the test condition, as with vulcanized rubber. Moreover, new mode relaxations can also be observed on random copolymers and B-4A-PIP, as indicated by red arrows which are assigned as α‘-relaxation. This may be aroused by the terminal interactions. 

**Figure 3:**
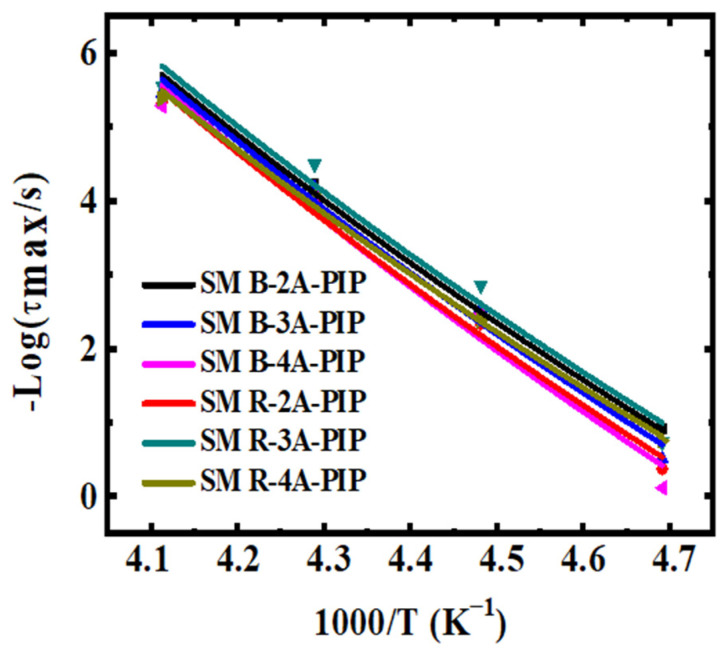
Temperature dependence of *τ*max for SM relaxation of different samples. Solid lines represent fitting curves.

For more details, quantitative analyses are carried out in the frequency domain. The typical dielectric loss spectra are shown in Figure S11. A clear loss peak emerging around −50 °C (indicated by blue arrows in Figure S11) should be due to SM relaxation [45]. This SM relaxation peak shifts to higher frequency with increasing temperature, which is known as a thermally activated process. The frequency spectra are on further fitted using the Havriliak–Negami function. The temperature dependences of *τ*_max_ are shown in Figure 3. However, no obvious differences in the SM relaxation speed are observed among these systems. Therefore, it is rational that the terminal oligopeptide and its length have weak influence on the segmental motions.

**Figure 4:**
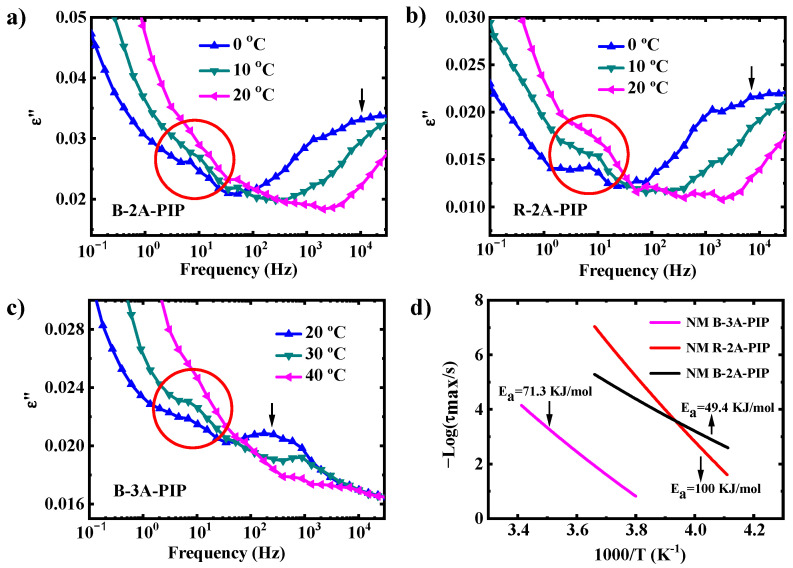
Dielectric loss *ε*″ as a function of the frequency at for (**a**) B-2A-PIP, (**b**) R-2A-PIP and (**c**) B-3A-PIP. (**d**) The temperature dependence of *τ*max for NM relaxation of B-2A-PIP, R-2A-PIP and B-3A-PIP.

Quantitative analyses are also carried out for NM relaxations. The typical dielectric loss spectra of B-2A-PIP, R-2A-PIP and B-3A-PIP at higher temperature are shown in Figure 4 and the NM relaxations are indicated by black arrows. The temperature dependence of *τ*_max_ can also be well described with Vogel–Fulcher–Tamman (VFT) equation. The NM relaxation speed of B-2A-PIP is slower than R-2A-PIP above 254 K, revealing the constraint of terminal blocks on longitudinal chain mobility in the system. Additionally, B-3A-PIP possesses much slower NM relaxation speed than B-2A-PIP. Conclusively, the increased oligopeptide length elevates the temperature of NM relaxation and decreases its speed which may be related to the different stability of β-sheets structures.

Furthermore, while the NM relaxation of B-2A-PIP, R-2A-PIP and B-3A-PIP conform to the VFT equation, the Arrhenius-like equation is used to calculate the apparent activation energy [48,49]:

(4) $$\tau =\tau_{0}\exp\left(\frac{E_{a}}{RT}\right)$$

where *E_a_* is the activation energy and *τ*_0_ is a proportionality constant. As marked in Figure 4d, the *E_a_* of the NM relaxation of B-2A-PIP is 49.4 KJ/mol, while that of B-3A-PIP increases to 71.3 KJ/mol. In fact, the NM process, detectable because of the dipole component parallel to the backbone, corresponds to motions of the entire chain. It indicates that the entire chain relaxation in B-3A-PIP is more severely constrained. The result corresponds well with the X-ray diffraction experiments, which shows the peptide secondary structures in B-3A-PIP are more ordered and compact than those of B-2A-PIP. Therefore, the oligopeptide aggregates in B-3A-PIP are more effective in anchoring the molecular chains, and thus the entire chain relaxation is more severely restricted. In addition, the activation energy of R-2A-PIP is the largest, reaching 100 KJ/mol. This finding confirms that the distribution of oligopeptides has a great influence on chain relaxation. From the secondary structure analysis by X-ray diffraction experiments (Figure 1d), we can find that the peptides in random copolymers tend to form loose aggregates. However, the number of loose aggregates far exceeds that of the more compact aggregates because hydrogen bonds between loose aggregates are more likely to form. Therefore, 2A-PIP possesses more non-covalent crosslinking, which results in the highest NM relaxation activation energy. In addition, compact aggregates are more stable, while the proportion of compact aggregates in R-2A-PIP is relatively small. Therefore, the relaxation time of R-2A-PIP is faster when the temperature is higher than 254 K, as shown in Figure 4d. For R-3A-PIP, B-4A-PIP and R-4A-PIP, the impacts of terminal oligopeptide on the NM relaxation temperature and speed are unclear due to the disappearance of the NM relaxation.

**Figure 5:**
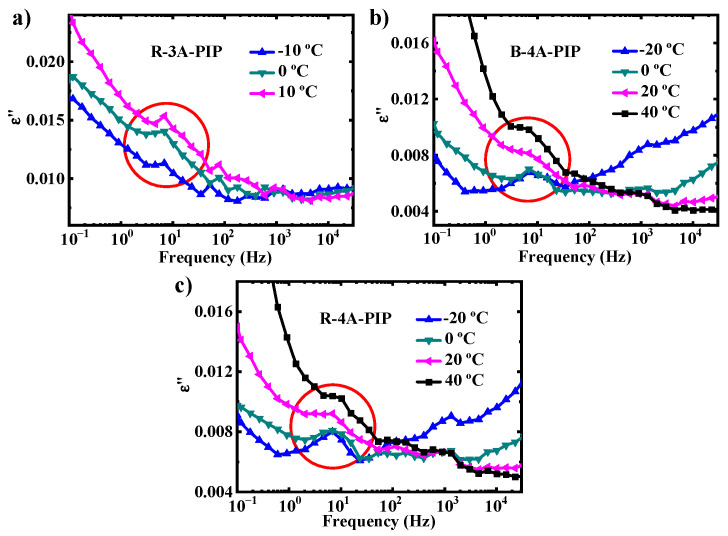
Dielectric loss *ε*″ as a function of the frequency for (**a**) R-3A-PIP, (**b**) B-4A-PIP and (**c**) R-4A-PIP.

**Figure 6:**
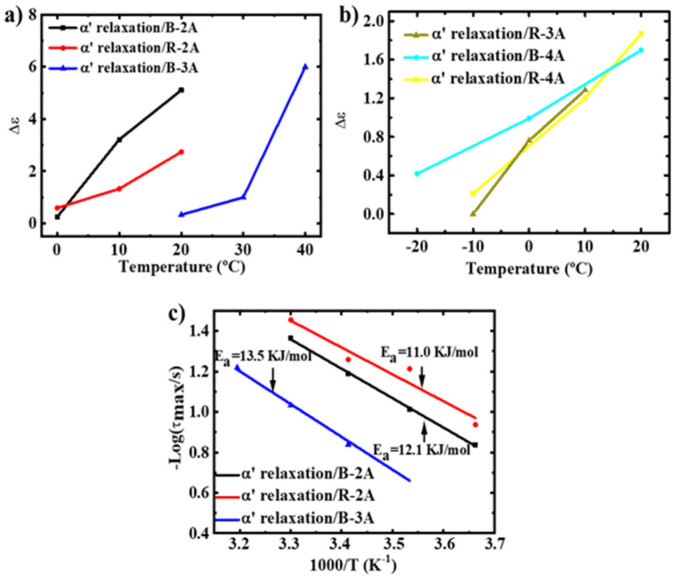
(**a**,**b**) Temperature dependence of ∆*ε* for a′ relaxation; (**c**) Arrhenius model for a′ relaxation. Solid line represents fitting curve.

Interestingly, in the typical dielectric loss spectra of all the samples, there are new peaks in the frequency range of 100–102 Hz, as indicated by red circles in Figure 4 and Figure 5. New relaxation processes are named as B-2A-α′ relaxation, R-2A-α′ relaxation, B-3A-α′ relaxation, R-3A-α′ relaxation, B-4A-α′ relaxation and R-4A-α′ relaxation for B-2A-PIP, R-2A-PIP, B-3A-PIP, R-3A-PIP, B-4A-PIP and R-4A-PIP, respectively. To clarify the origin of α′-relaxation, their dielectric strengths (Δε) are extracted from dielectric spectra and compared with NM relaxation. For polyisoprene, the increased temperature leads to decreased Δε of NM relaxation [50]. The Δε of NM relaxation of B-2A-PIP, R-2A-PIP and B-3A-PIP are also decreased with temperature elevation, as shown in Figure S12. Additionally, a slight decrease in Δε with temperature elevation is characteristic, reflecting the decreasing cooperativity of SM process at higher temperature [51]. However, the Δε of α′-relaxation exhibits the opposite trend with increasing temperature (Figure 6a,b). Thus, α′-relaxation is neither SM nor NM relaxation. Muller et al. [18] reported that functionalized polybutadiene with a small number of 4-phenyl-1,2,4-triazoline-3,5-dione showed new relaxation mode which is ascribed to the dissociation of the complexes. Inspired by this view, we turn to analyze the secondary structures of alanine oligopeptides and it has been reported that the alanine oligopeptide is arranged in an anti-parallel manner in the secondary structure [52,53]. This anti-parallel arrangement results in a centrosymmetric structure and should not have any dipole moment. Therefore, oligopeptide aggregates exhibit very low dielectric strength. As the temperature rises, the oligopeptide aggregates gradually shift to the dissociated state, which breaks the centrosymmetric structure between oligopeptides. This process increases the dipole moment of the system and, therefore, increases the dielectric strength. Thus, it is reasonable to conclude that α′-relaxation originates from the dissociation of complexed oligopeptides. To further describe the α′-relaxation, we fitted the dielectric spectra with HN equations and extracted the characteristic relaxation time (*τ*_max_) in Figure 6c. The temperature dependence of *τ*_max_ of B-2A-PIP, R-2A-PIP and B-3A-PIP can be well described with Vogel–Fulcher–Tamman (VFT) equation. Compared with NM relaxation, the α′-relaxation speed of B-2A-PIP, R-2A-PIP and B-3A-PIP is slower by several orders of magnitude, all close to 0.1 s. While B-2A-a′ relaxation, R-2A-a′ relaxation and B-3A-a′ relaxation conform to the VFT equation, the Arrhenius-like equation is used to calculate the apparent activation energy (*E_a_*). As shown in Figure 6c, the *E_a_* of B-2A-a′ relaxation and R-2A-a′ relaxation is, respectively, 12.1 KJ/mol and 11.0 KJ/mol, while the activation energy of B-3A-a′ relaxation increases to 13.5 KJ/mol. Strikingly, α′-relaxation peaks of R-3A-PIP, B-4A-PIP and R-4A-PIP almost keep at the same frequency with increasing temperature, which should be ascribed to the restricted mobility of entire chains. The *τ*_max_ of R-3A-PIP, B-4A-PIP and R-4A-PIP are temperature-independent, which are 0.151 s, 0.143 s and 0.146 s, respectively. Considering the similar activation energy of B-2A, R-2A and B-3A and similar *τ*_max_ of all six samples, it is speculated that the relaxation of peptide aggregates are closely related to the disentanglement of polyisoprene chains, which are quite independent on the length or distribution of oligopeptides. 

### 3.3. Classical Molecular Dynamics Simulation

**Figure 7:**
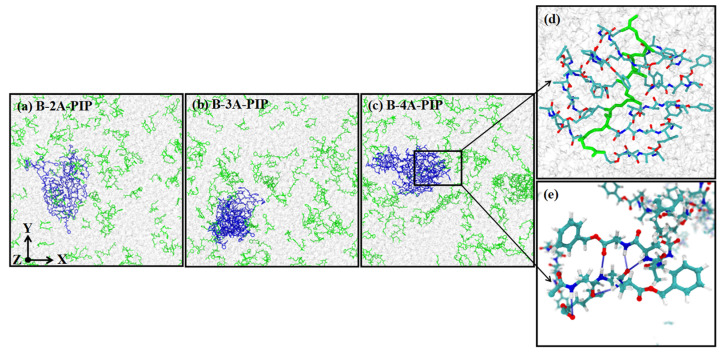
(**a**–**c**) Three different views of frame structures of B-2A-PIP, B-3A-PIP and B-4A-PIP from top with axis denoted figures. A closed black dot indicates that the axis is pointing into the paper. The block segment with 40 repeat units is colored in blue, while the random units are colored in green. PIP units are colored in light grey for clarity. All hydrogen atoms are hidden. (**d**) A part of the block segment of B-4A-PIP. (**e**) A typical random-tetrapeptide interacting units with hydrogen bonds shown in blue dotted lines.

To deeply understand the influence of terminal oligopeptide and its length on the microstructures of copolymers, the molecular dynamics simulation method was carried out. The frame structures of B-2A-PIP, B-3A-PIP and B-4A-PIP are shown in Figure 7. Oligopeptides in block part tend to form nano-sized aggregates, reflecting the influence of terminal oligopeptide on their self-assembly. The structures are further dissected into different layers along the z-axes for better understanding the distribution of oligopeptides and how they interact, as shown in Figure S13–S15. Block oligopeptides are surrounded by abundant random oligopeptides. For B-2A-PIP, the random dipeptides tend to be evenly distributed in all slices. For B-3A-PIP and B-4A-PIP, tripeptides or tetrapeptides increase in the slices with block oligopeptides presented. In particular, one more nano-sized aggregate formed by random tetrapeptides in Figure S14e also increase the number of tetrapeptide chains in that slice. These results can qualitatively support the experimental findings that the microstructures of copolymers become more heterogeneous with increasing oligopeptide length, and may increase the mechanical strength of elastomers. Detailed analyses into structural features of B-4A-PIP shown in Figure 7d,e indicate that block tetrapeptides are likely to form structures with obviously β-sheet feature. In comparison, the interactions between random tetrapeptides can form less ordered β-sheet features.

**Figure 8:**
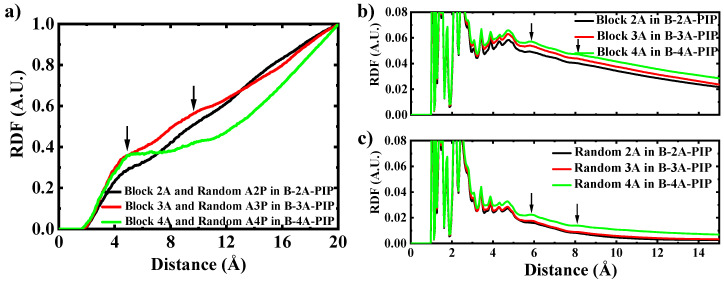
(**a**) The radial distribution functions between block-oligopeptides and random-oligopeptides; (**b**) The radial distribution functions for block-oligopeptides; (**c**) The radial distribution functions for random-oligopeptides.

The functions of terminal oligopeptide in copolymers are explored by putting block and random oligopeptides into one or two simulation boxes. Radial distribution functions (RDFs) are used to describe the structures, as shown in Figure 8. Firstly, the interactions between the block and random oligopeptides in one simulation box are analyzed (Figure 8a). In the tetrapeptide system, the peaks formed around 5 Å and 10 Å are sharper than those in the dipeptide and tripeptide systems, as indicated by the black arrows. This suggests that much more ordering structures are formed for tetrapeptides than the others. Increasing the oligopeptide length facilitates more ordering structures. Next, the interactions within block or random oligopeptides in two different simulation boxes are analyzed. In the block system, the curve with similar peak shapes but higher ordinate values are observed for tetrapeptides compared to dipeptides and tripeptides, as indicated by the black arrows. This suggests that increasing the oligopeptide length has a slight influence on the ordered degree of structures while significantly increasing the number of ordered structures in the block system, while in the random system, much sharper and higher peaks of tetrapeptides than the others are observed. It indicates that increasing the oligopeptide length improves both the ordered degree of structures and the number of ordered structures in the random system. Conclusively, the terminal block interaction increased the ordered structures which may well increase the mechanical properties while the increased length of oligopeptide form more, ordered sheet structures in random system. The two factors cause the abnormal difference of β-sheets structures in B-3A-PIP, R-2A-PIP and B-3A-PIP, R-3A-PIP.

## 4. Conclusions

Here, we introduce dipeptide, tripeptide and tetrapeptide into polyisoprene with different hydroxyl distributions, investigating the influence of oligopeptide length and distribution on the mechanical properties, molecular motion and self-assembly behaviors of polyisoprene. The increased oligopeptide length increases the ordered microstructure, which includes compact structure as well as loose structure, and the block distribution of oligopeptides can further increase the proportion of compact aggregates in the ordered microstructure, which is indicated by XRD. Chains dynamics probed by broadband dielectric spectroscopy (BDS) reveals the mechanism. The NM-relaxation and α′-relaxation speed of B-3A-PIP is slower than that of B-2A-PIP, while the *E_a_* of NM-relaxation and α′-relaxation of B-3A-PIP is higher than that of B-2A-PIP, demonstrating that the increased oligopeptide length enhances the restriction effect of oligopeptide aggregates on longitudinal chains movement by increasing the ordered microstructure. In addition, above 254 K, the NM relaxation rate of R-2A-PIP is faster than that of B-2A-PIP due to dissociation of the loose aggregates, demonstrating that there is indeed a larger proportion of compact aggregates in the block samples and, therefore, more severely restricted chain motion in the block samples. However, R-3A-PIP has no significant NM relaxation peak and its α-′ relaxation is temperature-independent. The mechanical properties of B-4A-PIP and R-4A-PIP are further improved compared with B-3A-PIP and R-3A-PIP. B-4A-PIP and R-4A-PIP also have no significant NM relaxation peaks and their α′-relaxations are temperature-independent. In the system with longer oligopeptide length, the mobility of longitudinal chains and oligopeptide complexes are significantly suppressed because of more stable β-sheets structures.

Herein, a new structure–properties relationship regarding terminal interaction in elastomer has been developed. The increased oligopeptide length improves the ordered degree of structures and the number of ordered structures in the matrix which are closely related to the mechanical properties. The binding strength of aggregates derived from the terminal blocks is remarkably improved, which dominantly impacts on the dynamic properties. The mobilities of longitudinal chains and oligopeptide complexes are gradually suppressed. The reinforced robustness of networks ultimately increases the mechanical properties of polyisoprene.

## Data Availability

The data presented in this study are available on request from the corresponding author.

## Supplementary Materials

The following are available online at https://www.mdpi.com/article/10.3390/polym13244408/s1, Experimental procedures, analytical data and additional spectra. Figure S1. ^1^H NMR spectrum of B-DSC-PIP; Figure S2. ^1^H NMR spectrum of B-2A-PIP; Figure S3. ^1^H NMR spectrum of B-3A-PIP; Figure S4. ^1^H NMR spectrum of R-DSC-PIP; Figure S5. ^1^H NMR spectrum of R-2A-PIP; Figure S6. ^1^H NMR spectrum of R-3A-PIP; Figure S7. Representative pictures of samples. All samples show the same appearance; Figure S8. Synthesis of B-2A-PIP, R-2A-PIP, B-3A-PIP and R-3A-PIP; Figure S9. Stress–strain curves as a function of strain of PIP, B-OH-PIP and R-OH-PIP; Figure S10. (a) Stress–strain curves as a function of strain; (b) FTIR spectra and (c) X-ray diffraction spectra of B-4A-PIP, R-4A-PIP; Figure S11. Dielectric loss ε″ as a function of the frequency at −60 °C to −30 °C for different copolymers: (a) B-2A-PIP, (b) B-3A-PIP, (c) B-4A-PIP, (d) R-2A-PIP, (e) R-3A-PIP, (f) R-4A-PIP; Figure S12. The temperature dependence of Δε for NM relaxation of B-2A-PIP (a), R-2A-PIP (b) and B-3A-PIP (c); Figure S13. Three different views of last frame structures of B-2A-PIP from top, side 1 and side 2 with axis denoted figures. A closed black dot indicates such axis is pointing into the paper while the open dot suggests the axis is pointing outwards. The block segment of 2A with 40 repeat units is colored in blue, while the random units are colored in green. PIP units are colored in light grey for clarity. All hydrogen atoms are hidden. We plotted structures for slices along the z-axis. Each slice has a thickness of 2 nm (with Figure S1f slightly greater than 2 nm); Figure S14. Three different views of last frame structures of B-3A-PIP from top, side 1 and side 2 with axis denoted figures. A closed black dot indicates such axis is pointing into the paper while the open dot suggests the axis is pointing outwards. The block segment of 3A with 40 repeat units is colored in blue, while the random units are colored in green. PIP units are colored in light grey for clarity. All hydrogen atoms are hidden. We also plotted structures for slices along the z-axis. Each slice has a thickness of 2 nm (with Figure S1f slightly greater than 2 nm); Figure S15. Three different views of last frame structures of B-4A-PIP from top, side 1 and side 2 with axis denoted figures. A closed black dot indicates such axis is pointing into the paper while the open dot suggests the axis is pointing outwards. The block segment of 4A with 40 repeat units is colored in blue, while the random units are colored in green. PIP units are colored in light grey for clarity. All hydrogen atoms are hidden. We also plotted structures for slices along the z-axis. Each slice has a thickness of 2 nm (with Figure S3f slightly greater than 2 nm).
