# Supplementary material for: Influence of Oligopeptide Length and Distribution on Polyisoprene Properties
Source: Polymers (Basel). 2021 Dec 15;13(24):4408. doi: 10.3390/polym13244408 (PMC8708911; doi:10.3390/polym13244408)
Supplement: Supplementary file 1 [file polymers-13-04408-s001.zip › polymers-1492664-supplementary.pdf]

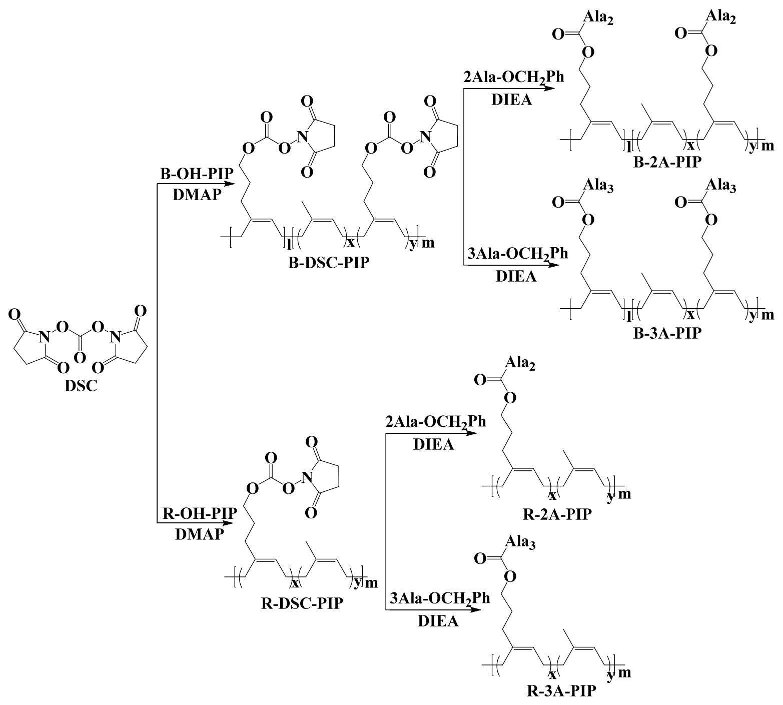

Figure S1. Synthesis of B-2A-PIP, R-2A-PIP, B-3A-PIP and R-3A-PIP.

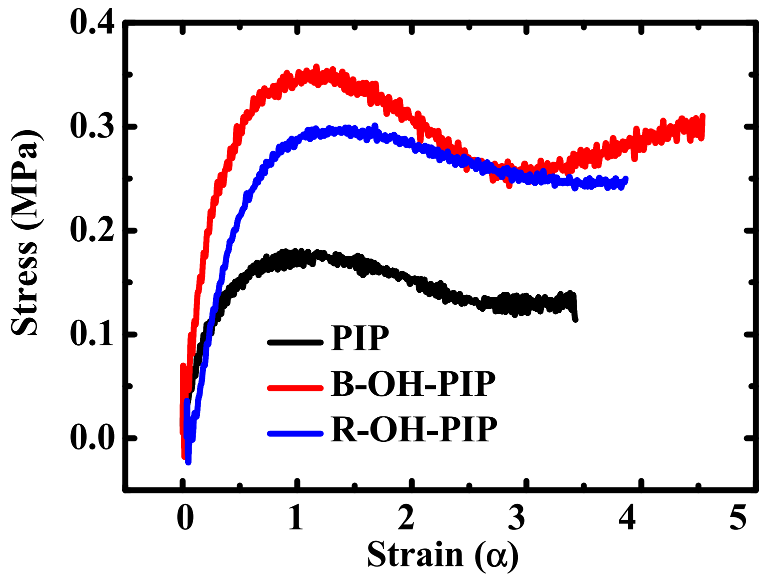

Figure S2. Stress–strain curves as a function of strain of PIP, B-OH-PIP and R-OH-PIP.

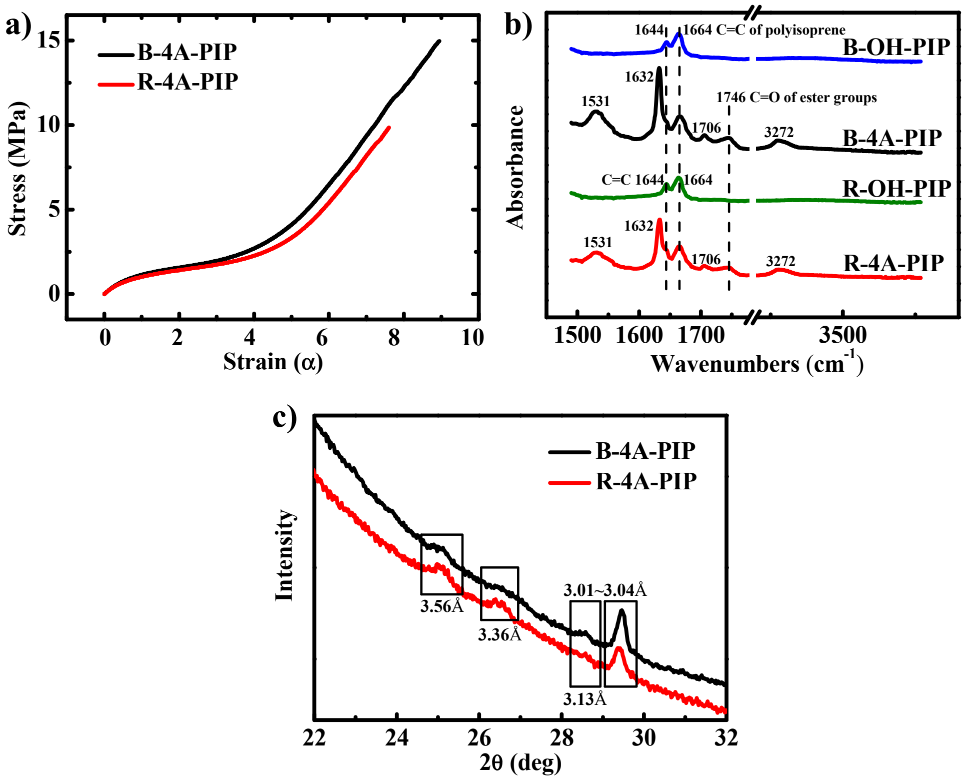

Figure S3. (a) Stress–strain curves as a function of strain; (b) FTIR spectra and (c) X-ray diffraction spectra of B-4A-PIP, R-4A-PIP.

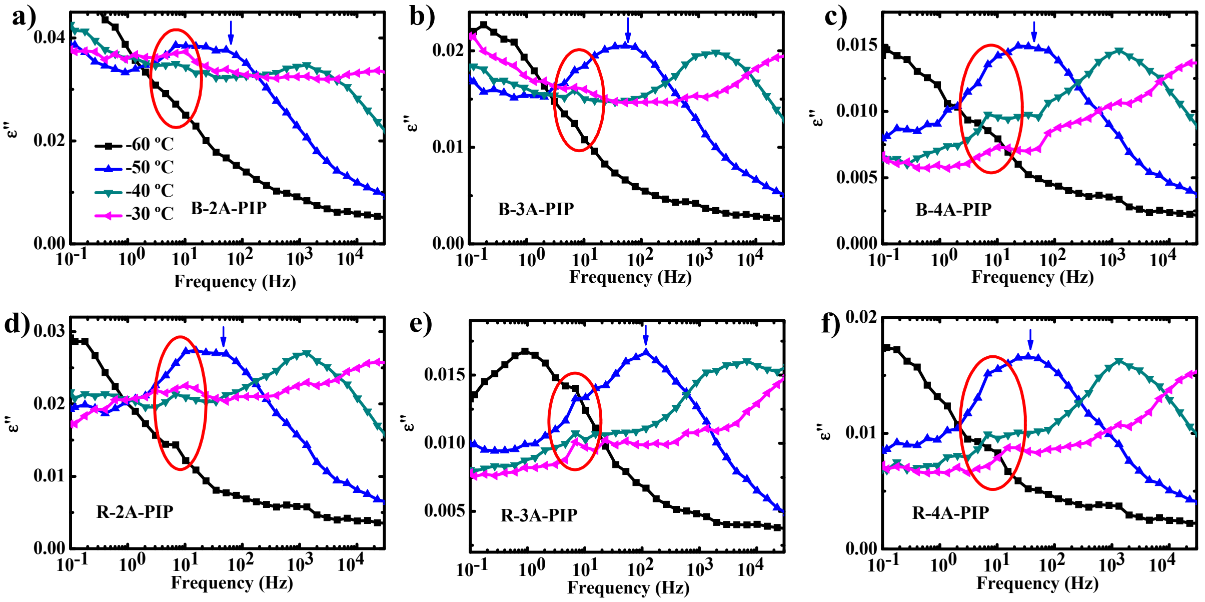

Figure S4. Dielectric loss ε″ as a function of the frequency at -60 ºC to -30 ºC for different copolymers: (a) B-2A-PIP, (b) B-3A-PIP, (c) B-4A-PIP, (d) R-2A-PIP, (e) R-3A-PIP, (f) R-4A-PIP.

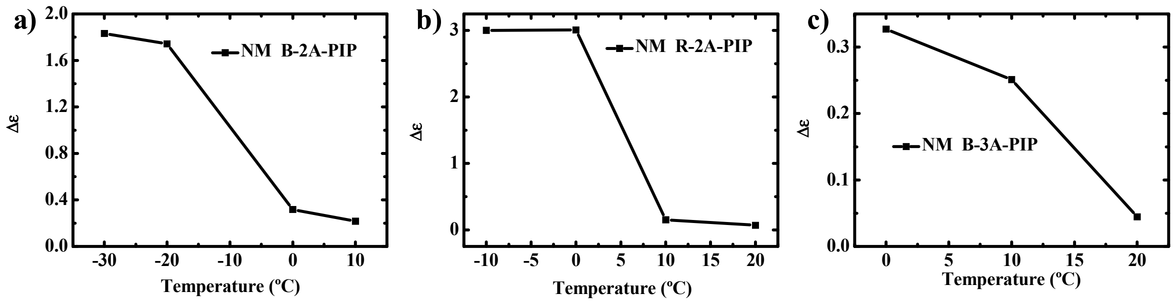

Figure S5. The temperature dependence of Δε for NM relaxation of B-2A-PIP (a), R-2A-PIP (b) and B-3A-PIP (c).

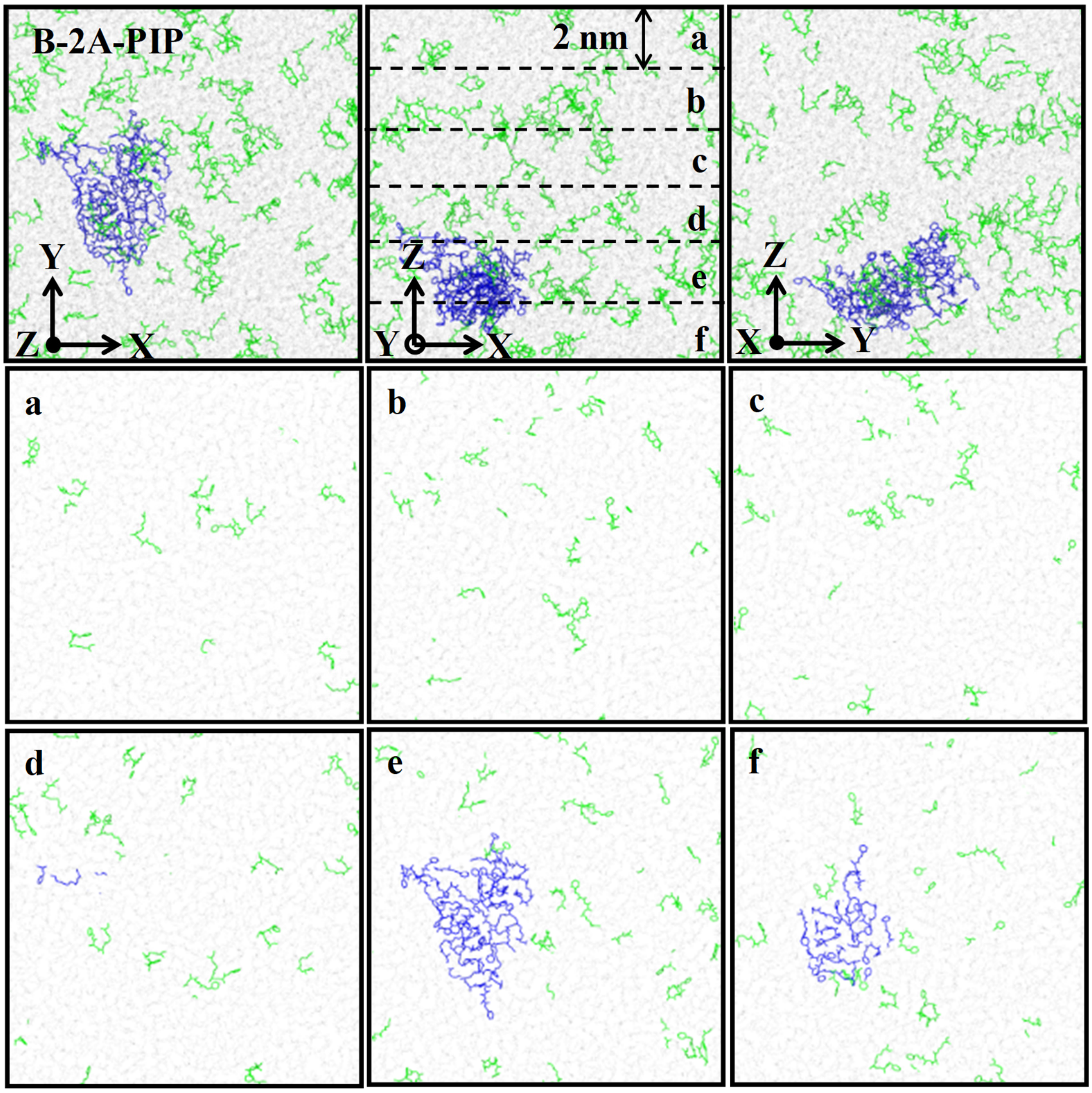

Figure S6. Three different views of last frame structures of B-2A-PIP from top, side 1 and side 2 with axis denoted figures. A closed black dot indicates such axis is pointing

into the paper while the open dot suggests the axis is pointing outwards. The block segment of 2A with 40 repeat units is colored in blue, while the random units are colored in green. PIP units are colored in light grey for clarity. All hydrogen atoms are hidden. We plotted structures for slices along the z-axis. Each slice has a thickness of 2 nm (with Figure S1f slightly greater than 2 nm).

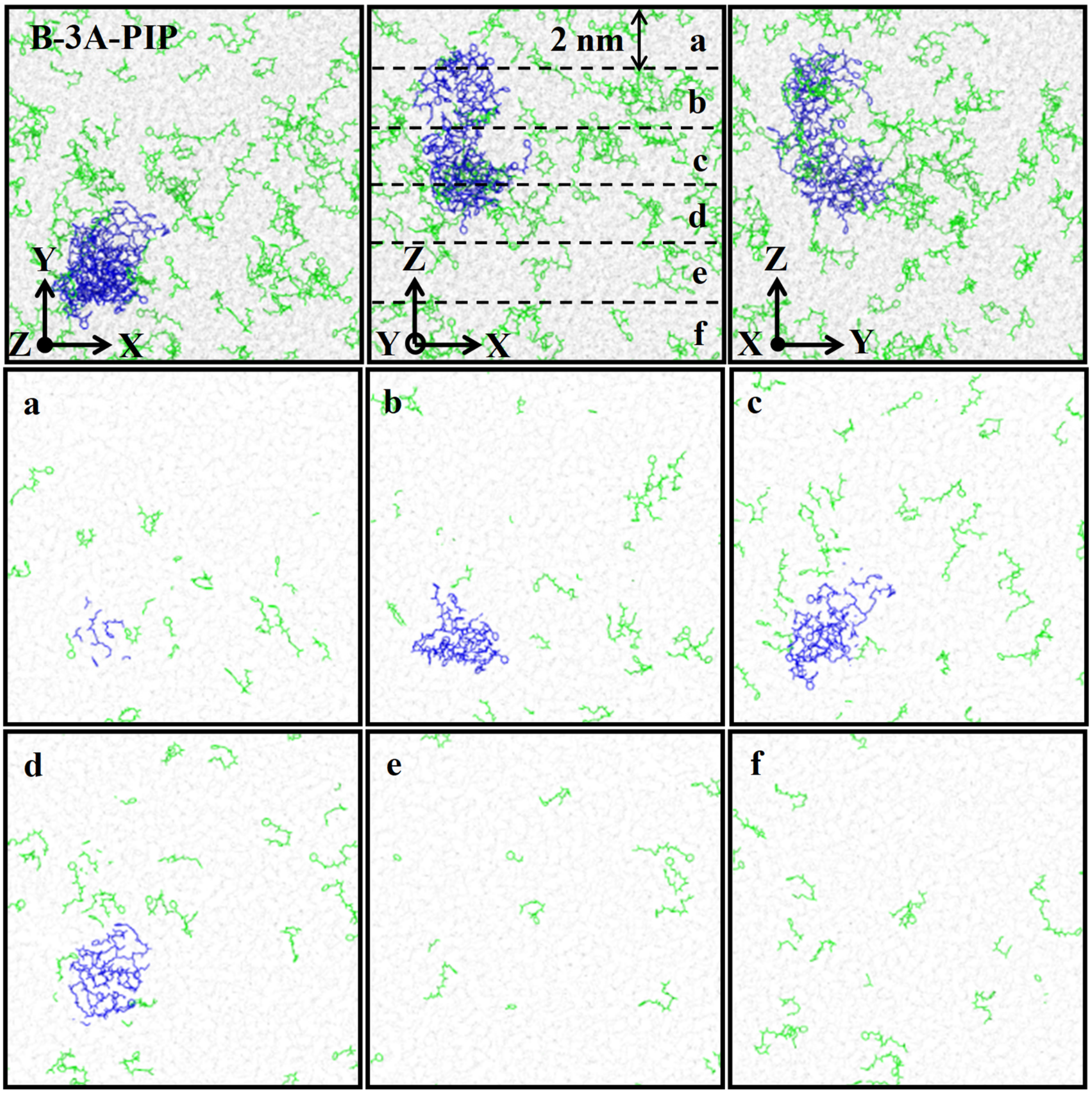

Figure S7. Three different views of last frame structures of B-3A-PIP from top, side 1 and side 2 with axis denoted figures. A closed black dot indicates such axis is pointing into the paper while the open dot suggests the axis is pointing outwards. The block segment of 3A with 40 repeat units is colored in blue, while the random units are colored in green. PIP units are colored in light grey for clarity. All hydrogen atoms are hidden. We also plotted structures for slices along the z-axis. Each slice has a thickness of 2 nm (with Figure S1f slightly greater than 2 nm).

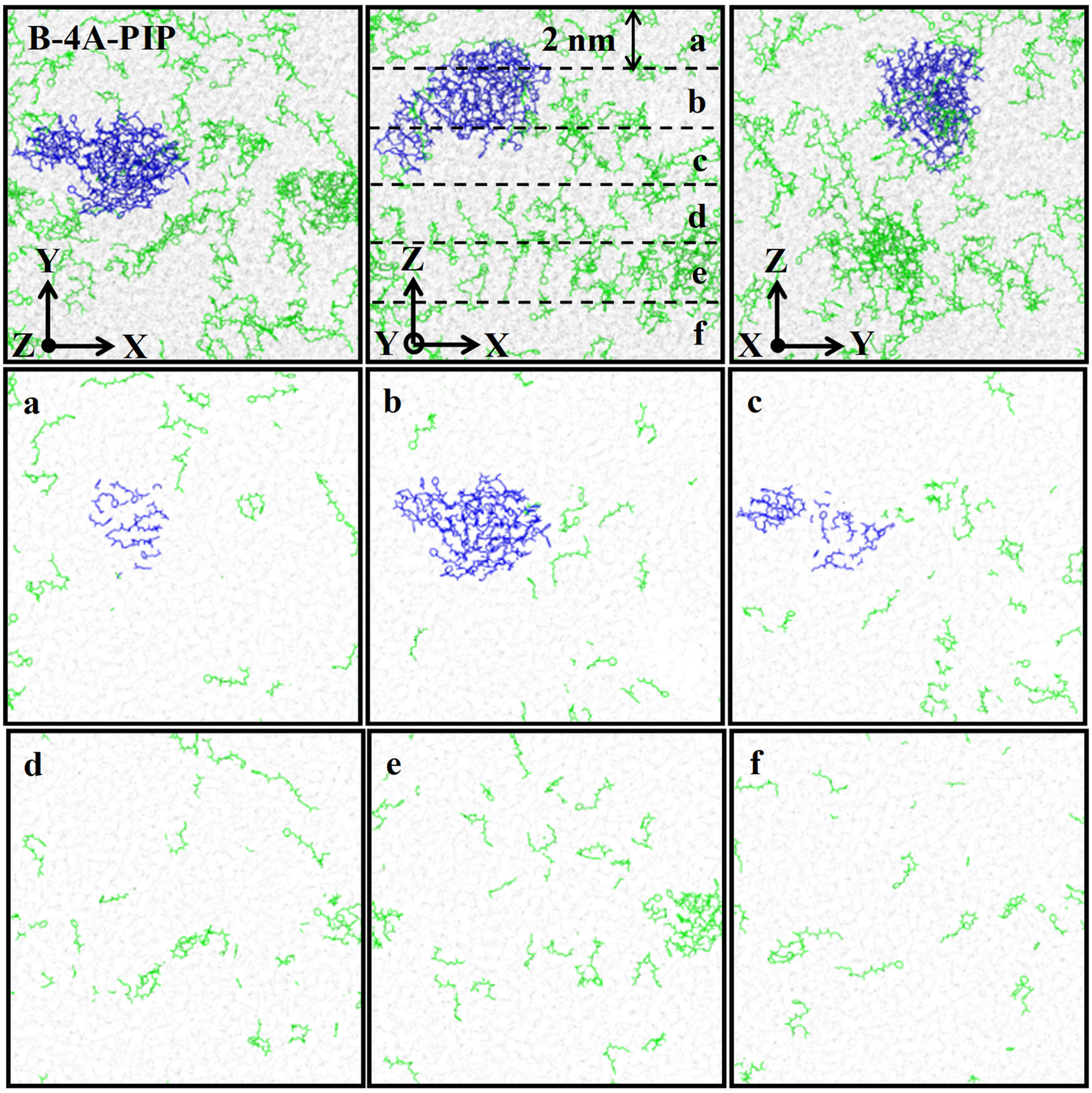

Figure S8. Three different views of last frame structures of B-4A-PIP from top, side 1 and side 2 with axis denoted figures. A closed black dot indicates such axis is pointing into the paper while the open dot suggests the axis is pointing outwards. The block segment of 4A with 40 repeat units is colored in blue, while the random units are colored in green. PIP units are colored in light grey for clarity. All hydrogen atoms are hidden. We also plotted structures for slices along the z-axis. Each slice has a thickness of 2 nm (with Figure S3f slightly greater than 2 nm).

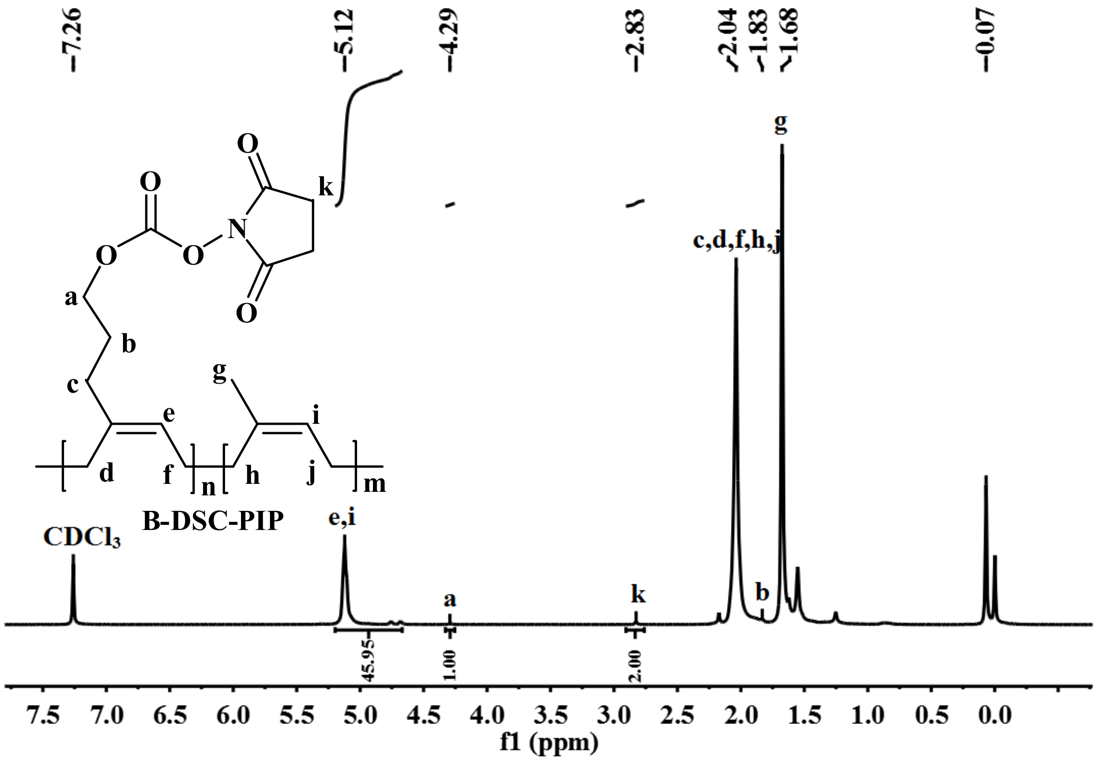

Figure S9. ^{1}H NMR spectrum of B-DSC-PIP.

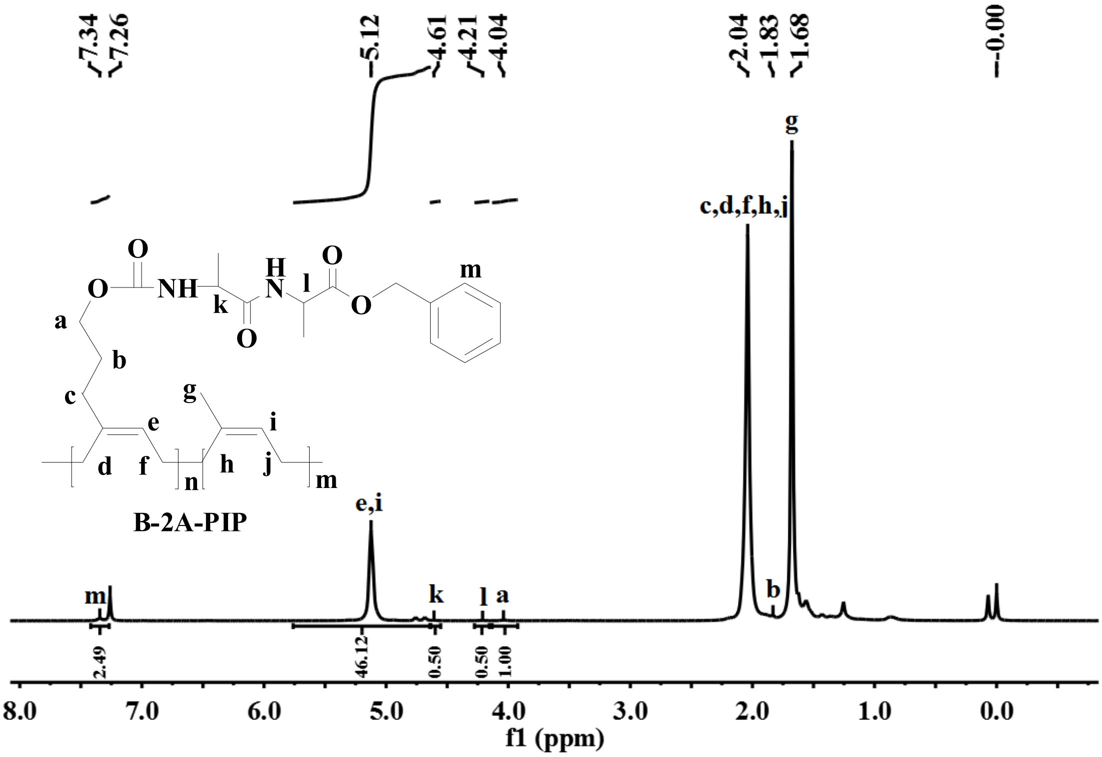

Figure S10. ^{1}H NMR spectrum of B-2A-PIP.

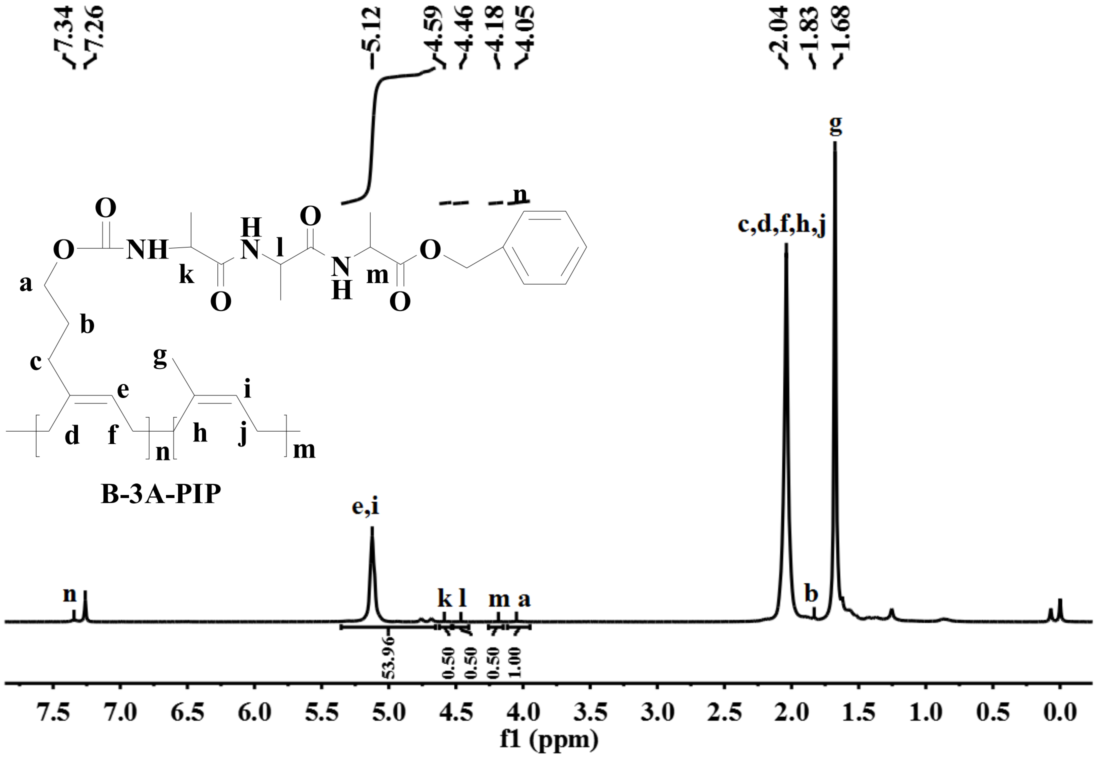

Figure S11. ^{1}H NMR spectrum of B-3A-PIP.

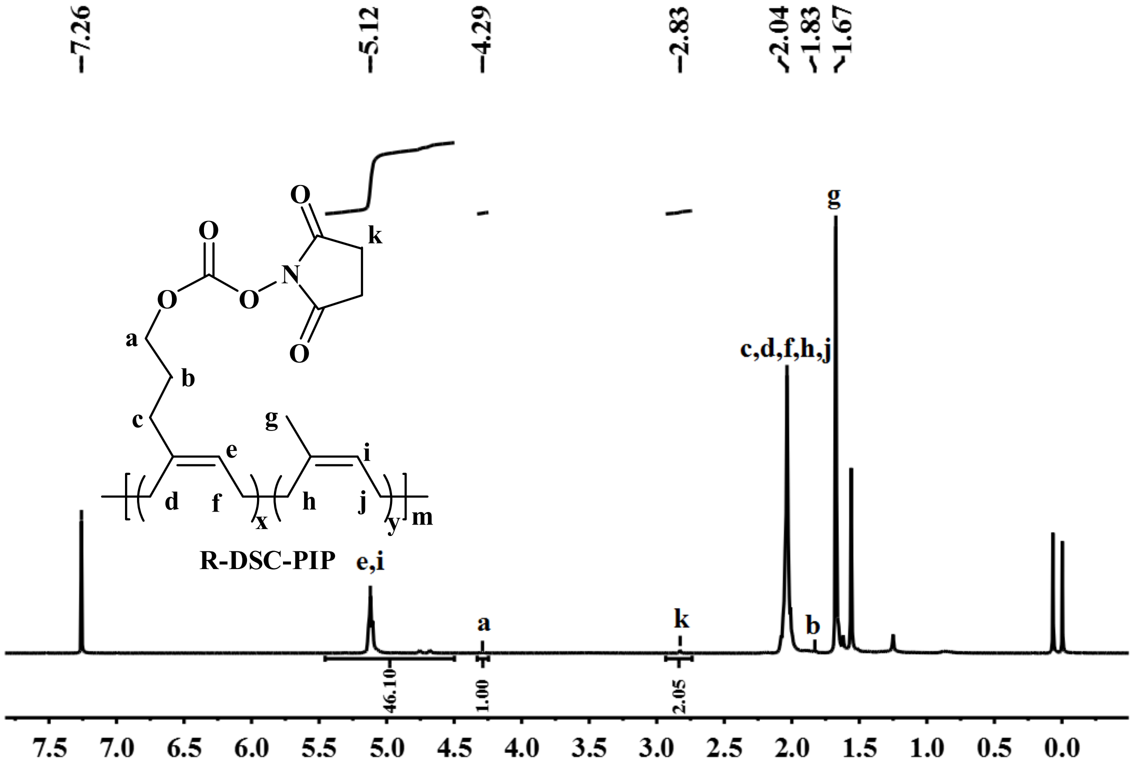

Figure S12. ^{1}H NMR spectrum of R-DSC-PIP.

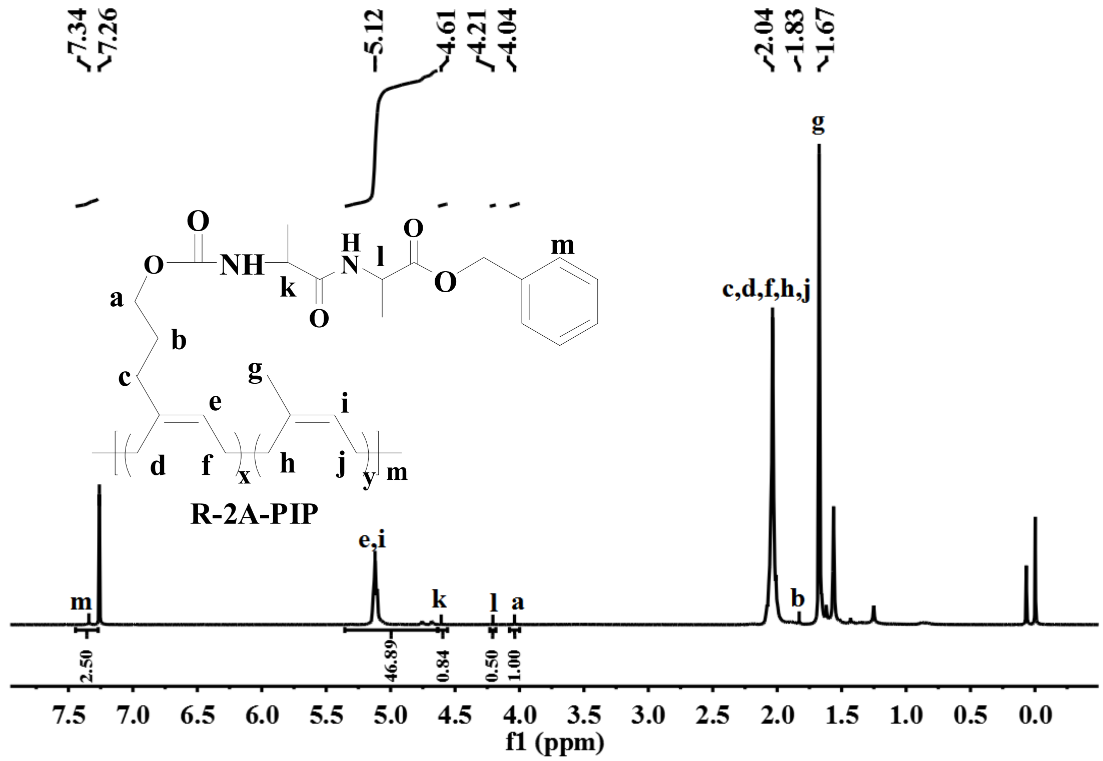

Figure S13. ^{1}H NMR spectrum of R-2A-PIP.

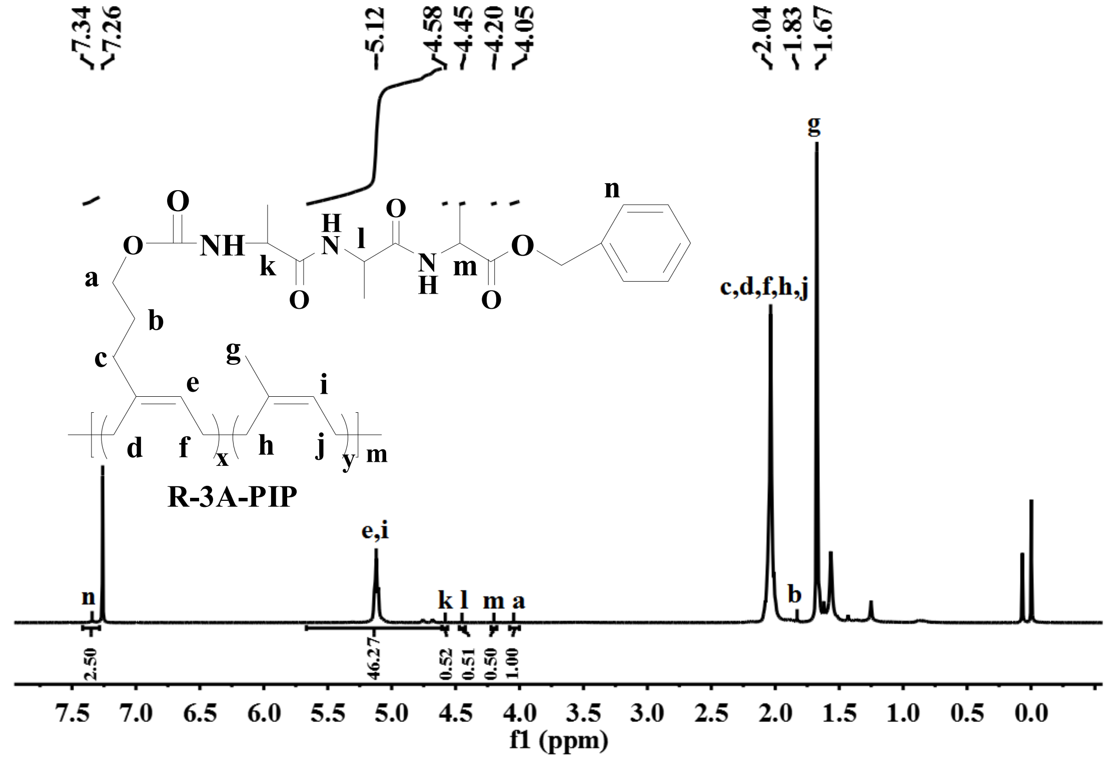

Figure S14. ^{1}H NMR spectrum of R-3A-PIP.

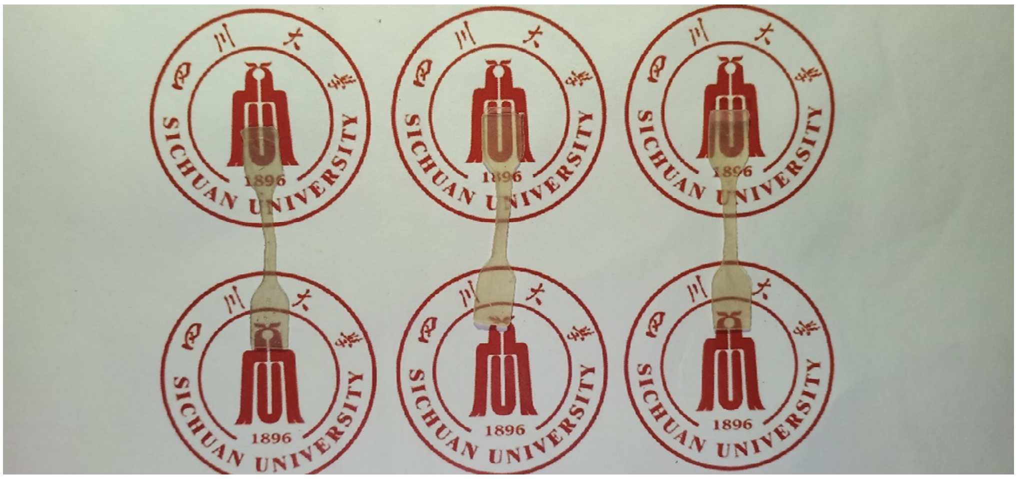

Figure S15. Representative pictures of samples. All samples show the same appearance.
